# Beliefs about people with albinism in Uganda: A qualitative study using the Common-Sense Model

**DOI:** 10.1371/journal.pone.0205774

**Published:** 2018-10-12

**Authors:** Caroline Bradbury-Jones, Peter Ogik, Jane Betts, Julie Taylor, Patricia Lund

**Affiliations:** 1 School of Nursing, University of Birmingham, Birmingham, United Kingdom; 2 Head Office, Source of the Nile Union for Persons with Albinism (SNUPA), Jinja, Uganda; 3 Advantage Africa, Olney, Buckinghamshire, United Kingdom; 4 Birmingham Women’s and Children’s Hospital NHS Foundation Trust, Birmingham, United Kingdom; 5 School of Life Sciences, Albinism in Africa project, Coventry University, Coventry, United Kingdom; St. George's University of London, UNITED KINGDOM

## Abstract

Albinism includes a group of inherited conditions that result in reduced melanin production. It has been documented across the world, with a high frequency in sub-Saharan Africa. There is very little published research about the lives of people with albinism, but available evidence shows that myths abound regarding their condition. They are feared, viewed with suspicion and believed to have supernatural powers. In this study we explored the links between beliefs, myths, traditions and positive/negative attitudes that surround people with albinism in Uganda. The study was located philosophically within Ubuntu—an Afrocentric worldview—and theoretically within the Common-Sense Model of self-regulation of health and illness that originates from the work of Leventhal in 2003. This qualitative study took place in eight districts of Busoga sub-region, Uganda between 2015 and 2017. Data collection comprised eight group discussions and 17 individual interviews with a range of informants, capturing the viewpoints of 73 participants. Findings lend support to previous research, highlighting the life-time discrimination and disadvantage experienced by many people with albinism. It shows that there is still much to be done to address the pervasive and potentially harmful beliefs and misconceptions about people with albinism.

## Introduction

Albinism includes a group of inherited conditions characterised by congenital hypopigmentation of the hair, skin and eyes in the case of the oculocutaneous (OCA) types. The lack of protective melanin pigment makes those affected very sensitive to the damaging effects of sun, with risks to the skin ranging from sunburn and blisters, thickening and wrinkling of skin to life limiting skin cancer [1]. A retrospective study of 64 cases of skin cancer in-patients with albinism at a large hospital in Tanzania found that most (84%) were under the age of 40 years, with 8% as young as 11–20 years of age [2]. Albinism adversely affects vision with impacts such as involuntary nystagmus, photophobia, poor depth perception, strabismus (squint), poor visual acuity and refractive errors [3]. Visual impairment among people with albinism is associated with poor visual acuity, [4], that has considerable impacts. For example in a South African study over 85% of the children with albinism had less than 30% vision, even with optical correction [5].

Although found worldwide [6], OCA has a high frequency in populations in sub-Saharan Africa, at 1 in 1755 in the south west African country of Namibia and 1 in 2673 in Tanzania in east Africa [7]. The prevalence of albinism has been estimated to be in the region of 1 in 2000–5000 throughout sub-Saharan Africa [8–10]. Albinism is known to be widespread in Uganda, necessitating the creation of organisations such as Source of the Nile Union of Persons with Albinism (SNUPA). However, to date there have been no large-scale prevalence studies, nor any other sources of empirical data to capture the lives of people with albinism in the country. Hence, the need for the study reported in this article.

In Africa, lack of the usual dark pigmentation found in indigenous populations makes the visible appearance of those with the condition markedly different to those in their families and communities without albinism. They are in effect what Phatoli and colleagues [11] describe as ‘being black in a white skin’. This has significant, negative psychosocial and cultural impacts brought about by perceptions of ‘otherness’ [12, 13]. Their lives are often marred by stigmatisation and rejection, lack of acceptance and limited social integration [14, 15]. As a result of their perceived difference, people with albinism are feared and viewed with suspicion, while simultaneously considered to have mystical powers. There is a misconception that their body parts can bring good luck, success and easy wealth but on the other hand, they are believed to be a curse, bringing bad luck [1].

In extreme (but not infrequent) cases, superstitions and traditional beliefs about albinism can lead to violent assault and murder [13]. Body parts of those with albinism are used in witchcraft-related rituals that typically involve them being made into charms that are believed to bring wealth and good luck. Attacks take different forms, such as forcibly shaving off hair, mutilation of fingers, limbs, ears and genitalia, and murder. In sum, otherness poses a significant societal risk for people with albinism and a direct threat to their human rights [13]. Underpinning this are deep rooted, culturally embedded beliefs about people with albinism that simultaneously hold them as enigmatic and frightening. To date however, such beliefs have been understood largely through anecdote, rather than empirical investigation.

Of available evidence, an early study by Braathen and Ingstadt [16] in Malawi, found a range of myths associated with albinism and the authors reported multiple problems experienced by people with albinism as a result. Similarly, Baker and colleagues [17] found that beliefs about people with albinism in South Africa and Zimbabwe had a profound influence on people’s life. Their study showed how even when the genetic explanation for albinism is accepted, it runs parallel to existing beliefs. More recently, studies in Tanzania [12, 18] and South Africa [11] have extended the evidence base regarding the mythology of albinism. Importantly, they have highlighted its corollary; the endemic marginalisation of people with albinism.

Overall, there is a small but growing body of research conducted in sub-Saharan Africa that has highlighted the impacts of beliefs about people with albinism on their lives. The research emanates from Malawi, Tanzania, Zimbabwe and South Africa as countries that have been the primary focal point of research [6]. The issue of albinism in most other African countries has largely been ignored in the academic literature, or has only recently come to the fore, as is the case for example, with Uganda. Moreover, ways in which beliefs can be understood theoretically and philosophically remain under-developed. This article reports on the findings of a qualitative study conducted in Uganda that addressed this gap in evidence. The study was part of a larger project undertaken in Uganda and Tanzania that examined the stigma and fear surrounding albinism upon the education and life opportunities of children and young people directly affected by the condition. Exploring perceptions and beliefs held about the condition formed an important part of the study because it enabled any misconceptions about albinism that lead to discrimination and harmful practices to be challenged.

## Research question

We interrogated the Ugandan data to answer the following question: What are the beliefs that surround people with albinism in Uganda?

## Methodology

This was a qualitative study that draws on the Ugandan data only. It captures the viewpoints of a total of 73 participants via eight focus group discussions and 17 individual interviews. A range of informants took part, including people with albinism, parents of children with albinism, and people with expert and professional insights regarding the issue. The study, in eight districts of Busoga sub-region, Uganda took place between September 2015 and April 2017.

### Philosophical lens and theoretical framework

The study is located philosophically within the worldview of Ubuntu–described as an Afrocentric approach [19] and theoretically, we draw upon the Common-Sense Model (CSM) of self-regulation of health and illness that originates from the work of Leventhal [20].

#### The philosophical lens of Ubuntu

Globalisation has led to infiltration of Western knowledge systems into African communities that have resulted in the marginalisation and submergence of African indigenous knowledge systems [21]. Across the world, some researchers are attempting to address this colonial legacy and redress Western knowledge hegemony [22, 23]. In the context of sub-Saharan Africa, the decolonisation of research and the protection of indigenous knowledge has become an imperative [24–27]. A groundswell of ‘decolonising’ [27], ‘indigenous’ [28] and ‘Afrocentric’ [19, 29] methodologies has developed that are based on the same philosophy: the privileging of African indigenous knowledge and culture. They focus on cultural and social immersion as opposed to scientific distance as the best approach to understand African phenomena [29]. In identifying the features of such methodologies, researchers have suggested Ubuntu as an important worldview through which to investigate phenomena [26]; in our case, beliefs about albinism.

Seehawer [27] acknowledges that Ubuntu does not translate easily into Western languages, but in English it is most often interpreted as *I am*, *because we are*, which seeks to capture how in Africa, individuals exist through community and that what happens to the individual, happens to the whole community. Ubuntu is not an abstract principle, nor a set of rules; it is both a philosophy and way of life [30]. Ubuntu is a Bantu (indigenous peoples of central and southern Africa who speak Bantu languages) characteristic of relationships, which emphasises communal life, based on sharing, respect and compassion [21]. It is one in which the perspective of African people is centred, located and grounded, and it honours indigenous culture [22]. Ubuntu can be reflected in research designs through participatory approaches, which are at its core, with in-depth interviews, stories and songs as important ways of knowing in indigenous knowledge research [25]. Methods that engender story-telling and narrative as opposed to structured questions are appropriate because stories are a powerful indigenous methodology, and the conversation method shows respect for participants’ stories and provides them with greater control [28]. We conducted individual and group interviews that took account of this and allowed participants to tell their stories. For example, the individual interviews were open and flexible, with minimal direction and probing from the interviewer. The group interviews were somewhat different in style to the typical focus group interview in Western countries, that encourages group interaction and sometimes debate [31]. Our group interviews were akin to a ‘research-sharing circle’, described by Kovach [28], whereby participants shared their stories individually, listened to each other and waited patiently and respectfully for their turn in the story-telling circle.

#### The Common-Sense Model as a theoretical framework

Central to CSM are representations—or beliefs–that people have about illness. Leventhal and colleagues describe five elements of these representations that people may hold: identity (the label or name given to a condition); cause (ideas about perceived causes of a condition); consequences (perceptions regarding the consequences of a condition); timeline (beliefs about how long the condition will last) and control (beliefs about the extent to which a condition can be cured or controlled). Table 1 shows how we have interpreted these original understandings so that they meet the needs of the current study.

**Table 1:** Interpretation of CSM applied to this study.

Domain	Original understandings by Leventhal et al.	Interpretations in this study
Identity	The label given to a condition	Beliefs people hold about albinism in Ugandan society, particularly in rural areas
Cause	Ideas about perceived causes and associations	People’s explanations and misperceptions about the power of body parts of those with albinism that are capable of curing disease
Consequences	Perceptions regarding the consequences of a condition	How people talk about the consequences of albinism on the affected individual, the wider family and the community
Timeline	Beliefs about how long a condition will last	People’s descriptions of the impacts of albinism across the life-course (from birth through childhood to adulthood)
Control	Beliefs about the extent to which a condition can be cured	People’s beliefs about helpful interventions, practices and sources of support for those affected by albinism in Uganda

CSM is a complex, multi-level framework that has been used extensively in health research. It is typically used in disease/condition orientated research relating to how people’s beliefs influence their own behaviour. Two of the authors [CB-J and JT] have adapted CSM successfully in a previous studies exploring non-disease/illness related issues such as domestic abuse. We found that CSM lends itself well to focus on beliefs about an issue not only from those who experience it directly (i.e. those living with the condition/experience), but also those who are affected indirectly such as family members, professionals etc. This inspired us to draw on CSM again in the context of the current study, as a novel, theoretical framework through which to analyse the data. CSM provided opportunity for us to understand beliefs about albinism from multiple perspectives, not only those affected directly by the condition. Overall, the article makes a contribution not only empirically, but it also strengthens the theoretical and philosophical base for understanding this important and complex issue.

### Ethics

Ethical permissions were granted by the Research Ethics Committee at Coventry University (P32487) as the grant-holding institution and from the participating countries. In Uganda, written permissions were obtained from the Member of Parliament representing people with disability in Eastern Uganda. At the beginning of each focus group discussion and individual interview, the purpose of the study and the consent process were fully explained (in the local language if necessary) and each participant signed a consent form. Participant safety was imperative, particularly given the threat of discrimination and attack on many of those who took part. Choice of venue helped to mitigate the risks, along with the familiarity and relative protection associated with the SNUPA research team. We faced one particular conundrum. Many participants wanted their real names to be used in dissemination and did not want numbers, codes or pseudonyms. Although in tension with the Ubuntu philosophical underpinnings of the study that respects local norms, values and wishes, we privileged safety. It was too risky to reveal names and we have removed all identifying information, including whether participants took part in focus groups or individual interviews. We would prefer to be criticised for over-caution than risk the safety of participants.

### Group discussions in a sharing circle

A profile of the composition of the group discussions is provided in Table 2. The Ugandan research team (comprising PL as co-author and those individuals named in the acknowledgement statement) conducted eight group discussions each with between four and 12 participants (total of 56). These included two pilot group discussions that were included in the analysis as the quality of data was high. In other words, no revisions were made to the interview guide following the pilot phase (indicating that the questions and processes were appropriate) and moreover, the data were full and rich in content. Sites were purposively selected for their geographical and demographic variation, reflecting the distribution of people with albinism across the region. Five group interviews took place in rural communities (including island/lakeshore settings), two were in semi-urban communities, on the outskirts of small towns and one was in an urban setting. The group discussions took place in specifically hired rooms in community buildings such as schools because these were safe and familiar environments for most who took part.

**Table 2:** Group composition.

Data collection activity	Number of participants
Group 1 (pilot)	8
Group 2 (pilot)	12
Group 3	6
Group 4	8
Group 5	4
Group 6	5
Group 7	6
Group 8	7
**Total**	**56**

All participants, whether taking part in individual or group interviews (n = 73), were recruited via the Ugandan organisation SNUPA who were collaborators on the study. Each district has a SNUPA representative, a man or woman with albinism, who took responsibility for identifying and inviting participants to take part. Known and trusted by the participants, each district representative also played a role in facilitating their respective focus group discussion. Most group discussions lasted one hour and were conducted according to the following discussions points, presented here in English and the local language, Luganda:

Please can you tell me about your life as a person with albinism?
Osobola okunyumizako ebikwataganna no bulamubwo nga namagoye?
Tell me the story of when you were born
Mbulilako embozi ekwatagana nokuzalibwakwo
Tell me the story of your school life
Mbulilako obulamubwo nga olimusomero
What are the consequences of being a person with albinism?
Biiki bwoyisemu nga namagoye?
What has helped you most as a person with albinism?
Biiki ebisinze okuyamba gwe nga namagoye?

Interviews were conducted in Luganda by members of the SNUPA team and at the end of each group discussion the facilitators gave a summary of the events and thanked all participants for their contributions. Before leaving, each participant was provided with refreshments including a drink (soda) and chapatti or bread. They were also given funds to cover their transport costs.

### Individual interviews

Seventeen individual interviews (each lasting approximately one hour) were conducted with a range of people identified to have personal experience, knowledge and expertise of working with people with albinism. These included faith leaders, media representatives, academics and health professionals. Most were based in urban locations but had experience of working with people with albinism in all geographical settings. The SNUPA research team conducted the interviews and to ensure consistency across the study, the discussions followed the same questions as the group discussions. In line with the Ubuntu philosophy, an open, flexible approach was adopted that allowed participants’ stories to be told. Interviews were conducted in the local language (Luganda).

### Data analysis

The group discussions and individual interviews took place concurrently, with neither taking a more prominent role as regards informing the findings. Members of the local research team audio-recorded, translated from the vernacular and transcribed verbatim the group and individual interviews. All are fluent in both English and Luganda which meant that back-translation was considered unnecessary. During analysis we noted that a distinct feature of the group discussions was turn-taking, rather than the backward and forward discussion that is so typical of traditional focus groups. This reflected perfectly the norms of a research-sharing circle [28]. Data were analysed using a two-stage process. We did not use software for organising data analysis because we were concerned that this would exclude the Ugandan research team who did not have access to such resources. The first stage involved an inductive, thematic analysis of each transcript with the identification and labelling of initial codes that were then grouped to form rudimentary themes. Emergent themes were then identified across the whole data set by comparing and contrasting the themes from each transcript. This process was undertaken independently by two members of the UK research team [PL & SG], who met to agree the final themes in communication with the local team in Uganda [PO].

In the second analysis stage, a third member of the team [CB-J] mapped the inductively derived themes to the five domains of the CSM theoretical framework. Throughout the analysis process, communications across the team were frequent, instilling a team approach and agreement to the findings. For pragmatic reasons and specifically the geographical spread of those who took part, participants were not provided with opportunity to comment on the analysis. They do however have access to a summary of the findings and recommendations for Uganda, that is freely available at the SNUPA office.

## Findings

Findings are presented here under the CSM domains with illustrative quotes provided. Each theme was saturated and we had multiple quotes that we could have chosen for each. Restricted wordage means that we have not been able to include quotes from all 73 participants but we present quotations considered most meaningful and illuminative that also reflect a range of participant responses. The nature of qualitative research is that participants’ narratives are fluid, iterative, often disjointed and sometimes incomplete; they do not fit neatly into boxes. The rich stories that we were fortunate to capture, characterise the Ubuntu context within which the study was conducted. This means that there is some over-lap in the themes presented. We see this as a positive feature of our presentation of data that protects their meaning and minimises unnecessary reduction.

Within the albinism community there is debate about the use of the term ‘albino’, with the term ‘person with albinism’ preferred by many because it places the person first [32, 33]. We use this throughout except in direct quotation where people have used the term ‘albino’ themselves. We refer to ‘beliefs’ as those held by individuals, families and communities but through the lens of Ubuntu, the individual is always to be seen in the context of community.

### Identity

In the context of our findings, identity responds to the question ‘who are we? In our interpretation of CSM, it can also be taken to mean ‘who are you?’ Prominent beliefs among communities are that people with albinism are ghosts and that this is associated with great misfortune. Two people with albinism reported:

Persons in the community don’t appreciate persons with albinism because they say they are ghosts and when you get a ghost in the African circles, it means it’s a curse.

Many people misunderstand albinism; they always tell us after giving birth to children with albinism, “You have to do some ritual ceremonies to be cleansed from that curse”

Mothers recalled their own shock and that within their communities, of giving birth to a child with albinism. The second account illustrates how a medicalised, biological explanation is used in an attempt to dispel the myths:

The nurse saw the child coming out and shouted, “What’s this lady producing?” My husband’s relatives started complaining, “What has she produced”? Some said she produced misfortunes.

When I got my first child… I got shocked because there was no other child like that on the entire village; he was the first with albinism among all my children; community members always said I gave birth to lubaale (a ghost), after getting him, I took him to hospital, the doctors told me the child has no problem but his genes brought that colour… Community members said I produced a ghost.

The findings reveal a strong discourse of ‘otherness’ due to ghost and curse narratives that many participants challenged. In the following examples, one person with albinism (and the other without) talked of equality and normality and the issue of human rights:

We are the same people, we are one people; people should know we are the same people regardless of the sex, the colour, we are all created in God’s image, and we should share equal rights.

People living with albinism are actually normal people like us. They are actually very normal people like us, they can do everything like we do, they can go to school.

### Cause

Within this domain, we wanted to capture beliefs that respond to the question: what causes the birth of a child with albinism? It is closely linked with identity. Yet again, the ghost narrative was strong as illustrated by one mother (without albinism):

The moment I gave birth to [my baby daughter with albinism], they told my husband that a ghost impregnated me; I also remember one time I was passing by people playing some game, one gentleman told me to pass far from them, claiming that I slept with a ghost.

Cause attributed to the will of God was also evident as an act of punishment. An adult with albinism talked of demonic powers and linking with the identity domain, on how he was considered a spiritual being—a ghost–not a human being:

They thought that maybe my mother laughed at an albino somewhere in a certain society; it was God punishing her because she laughed. That’s what they told her. Others thought they did something bad and God punished them. It was from demonic powers and they did not believe that I am a human being; they thought I am a spiritual being.

Other participants held beliefs about causes that linked less to spiritual understandings and more to genetic explanations:

Giving birth to a person with albinism is hereditary; the mother and the father must have the gene of albinism. It is not a result of laughing at a person with albinism.

A mother gives birth to a child with albinism and the parents separate because the father of this child keeps saying the woman has the genes to produce albinism but the truth is both parents must be carriers so that they can produce a person with albinism.

### Consequences

Several participants without albinism talked of the consequences of fear, isolation and risk experienced by people with albinism that stem from mistaken beliefs among communities regarding their curative powers:

Very many people really fear people with albinism, because of what society has painted them; society thinks these people are outcasts, maybe ghosts, maybe they are not like us, they don’t have a skin colour like ours, everyone fears them.

These are beliefs even in today’s HIV/AIDS, that if you have sexual intercourse with a person with albinism, you are actually cured (of) HIV/AIDs.

I learnt from a family of five children that they were having sleepless nights because bad people wanted to steal these children and sacrifice them and get body parts… They tell you go and bring for me body parts, go and bring for me the ear of an albino.

A woman with albinism reflected on the isolation she experienced as a child; her narrative captured the misunderstandings of people within her community:

I was the only albino in the village but I wanted to play with fellow children. Each time I went to them, they ran away. They would run to their mothers. But being that I wanted to play with them, I chased them up to their parents. Their parents would chase me saying I could spread albinism to their children. Only few courageous people can accept to take the albino…Generally they fear us. Some people come to me and ask me weird questions; ‘Is it true albinos don’t eat fish?’

Two parents talked of their concerns about their children with albinism:

I always hear persons with albinism don’t die but just disappear. I have never seen a grave of an albino nor a dead body of an albino. I gave birth to her but am not sure whether she will just disappear… that’s all I wanted to know. I am afraid that maybe if [my daughter’s] time to die comes, she will just disappear.

Some of the children with albinism end up fearing other people because they know people want their hair or to kill them…. I have a lot of hatred about the people who want my children’s hair.

Among the stories of rejection and fear, there were examples of strength and resilience, illustrated here by the father of a child with albinism:

[On giving birth to my first born with albinism] Some people advised me to chase [leave] the wife but I kept on refusing since I love my wife. My wife later gave birth to the second child without albinism, so I continued being strong. After a year she gave birth to twins and both with albinism, so people again were shocked and stressed I should chase my wife. I asked myself where my wife would go with all those children, so I decided to just keep strong.

### Timeline

Timeline was a way of capturing the impact of beliefs about albinism throughout the life-course. Reports of being ostracised were common. Participants talked of their time in school where other children were concerned that albinism might be contagious, or teachers thought that children with albinism should be educated in separate schools:

Now when I would drink water in a cup my fellow pupils wouldn’t use the same cup again, if I eat food they would restrict them from using the same plate.

During my primary [school] we had a teacher who used to chase me out of class whenever she entered saying ‘you’re not supposed to be here’. I started thinking that maybe persons with albinism have their special schools. It also made me think that I am not treated as human as they are.

We found that employment and further education were difficult times for young people with albinism, who experienced discrimination at this point in their lives:

When I was looking for a job… one employer frankly said, ‘Why have they brought me that one? Will she manage to work with us?’… When I got a job, they never wanted to pay me, while some wanted to first sleep with me to pay me.

The biggest challenge I have faced is that most albinos never got the chance to go to school and it means they don’t have good jobs or white collar jobs

As with other aspects of our findings, in spite of the challenges, the consequences of living with albinism were not all negative. One 15-year-old boy with albinism informed us of his success in school:

In my primary [school] I used to behave very well even though my friends could mock on me. Sometimes [I was] advised to aspire for disciplinary prefect and when I just tried the votes came as if they were just waiting for me. I started leading while I was in primary six up to now where I am the academic prefect in our school.

One woman with albinism told us of her educational success, but there was ‘a sting in her tale’, serving as a reminder that discrimination is omnipresent:

I managed to study up to university level and graduated in development studies… Ever since I graduated, I have never got a job. Everywhere you go, people discriminate you. You go somewhere to request for the job and someone underestimates you thinking you can’t do anything… most of the people still discriminate us. Some don’t want to buy what we sell, just because an albino has touched the cloths.

### Control

In this study, control relates to beliefs about helpful interventions, practices and sources of support for people with albinism. One person with albinism captured the multiple adversities:

The biggest challenge we face is our lives; the sun affects us, it affects our eyes, the skin, some people isolate us but it has over the time reduced. In schools, some children are mistreated and end up dropping out of school. The situation is changing given that the government is now recognising us as people like the rest, the only difference being the skin colour. … I request the government to help these young children with albinism with sponsorships especially those from poor families.

Reinforcing the need for government support, one father of two children with albinism highlighted the challenge faced by parents, again illuminating the particular impacts on poorer families:

It’s a pity the government doesn’t help us [the parents of children with albinism]; I can at least educate my children but some parents can’t.

In the same way that people believed in God’s will as the cause of albinism, so too they had faith in God’s ability to protect them:

My parents were God fearing people and God helped them and they loved me as I was.

[I] am a happy courageous lady. Some people refuse us to walk during the night because of the high need for our hair, but I always say where there is God, there is no fear.

Although God and government featured highly in the data, many participants spoke of an influential other in their lives. Reflecting on the challenges to school children presented under the timeline theme, regarding control and positive intervention, teachers have a crucial role in support of children with albinism and in challenging misconceptions and acting as role models. One teacher without albinism explained her reaction to meeting a teacher with albinism:

Since I was introduced as a new teacher in the staff room, the teacher with albinism came close to me and wanted to shake hands with me; I hesitated and wondered whether I should really do it; I was asking myself how their hands feel; I later gave in my hand but in total fear; on greeting him, I realised the hand is just like mine.

The final quotes are from two prominent members of the community who both have albinism. The first explains the significant role of a teacher who had previously been discriminatory, but then changed his attitude:

My Dad talked to the teacher about ‘mocking my son and making him uncomfortable’. He said ‘You need to do something about this’. He (the teacher) started changing his way- he gave me a text book so I had my own and placed me in front of the class. Also told the students not to discriminate me, but unfortunately they already had the misperception about albinism so it became too difficult for them. But with time I started performing better than them. I was always number 1–5 among 150 students around seven years of age.

The words in this final quote capture the myriad beliefs about albinism that summarise our findings perfectly:

People actually have different concepts towards albinism. Some say it’s a curse, others say albinos are blessings, others say that when you sleep with an albino, you get wealthy, others (majority men and some few percentage of females) say that unprotected sex with an albino cures AIDS; others consider them spiritual persons. Some communities can even worship us. Therefore, different people have different perceptions towards the concept of albinism.

## Discussion

In many parts of Africa, the spiritual and present worlds are inter-twined and spirits are believed to be capable of influencing events in the present world [34]. It is important to understand this in interpreting the study findings. Ubuntu has provided a fitting, yet at time challenging, philosophical lens through which to explore beliefs about albinism. It is fitting in the sense that it privileges African knowledge systems and as Keane and colleagues [22] articulate, it ‘honours indigenous culture’. The stories that we heard therefore, are understood in a context where spiritual and present worlds connect and are equally ‘real’. From a Western viewpoint however, it can be difficult to make sense of the deep-rooted superstitions and myths associated with albinism. For example, as Franklin et al. [13] point out in their description of the killings of children with albinism to obtain their body parts for use in witchcraft-related rituals and charms, aside from the horror of these heinous crimes, the belief that they may have curative or other properties seems incomprehensible when considered against the science of Western medicine. But in the context of albinism in Africa, the fact that something might be scientifically impossible, is irrelevant to the social world [34]. It was the belief of one mother in our study that her daughter might not die, but just disappear. For her, this was a very real fear. It is with this in mind that the ‘common sense’ beliefs about albinism, brought to the fore in our study through the theoretical framework of CSM, are explored.

According to Baker and colleagues [17], myths are formed out of the need for human beings to make sense of unexpected and unanticipated events. Palmer [35] observed that the condition of albinism is surrounded by myths and this is supported by our study findings. Such misbeliefs lead to significant prejudice, stigma, antagonism and rejection [15, 36, 37]. This was evident when participants told stories about the consequences of being a person with albinism. Identifying and labelling people with albinism as ‘ghosts’ was a prominent theme. Supporting findings from Masanja et al. [18] and Brocco [12], our study highlighted the negative labels and terminology used to describe and define people with albinism. We were interested in beliefs–and probably more accurately misbeliefs—about the causes of albinism and again, our findings support those of previous studies. For example, in Tanzania albinism is misbelieved to be the result of a curse [14] or because a pregnant woman has looked at a person with albinism [16]. Other authors too, have explored the articulation in society that albinism is God’s will [11, 17, 38, 39].

While myths and misbeliefs abound about causes, some of our participants put forward biomedical explanations of albinism. These were however, from well-educated, activists and advocates, rather than reflecting the majority lay understandings. Similarly, in a qualitative study in Tanzania, Brocco [12] found that most community members had little knowledge of the biomedical explanations of albinism. As Baker and colleagues [17] argued, even when genetic explanations for albinism are accepted, they run parallel to existing beliefs.

Use of CSM encouraged us to view the experiences of people with albinism according to a timeline. Through this we were able to identify the significant disadvantage experienced by them throughout the life-course. Throughout their lives, people with albinism are at risk of violence and murder. The risk arises from a pervasive, deep-rooted misbelief about their curative powers. Through witchcraft related rituals, they are mutilated and killed, with their body parts used as powerful *muti* (medicine) for good luck, or turned into so-called ‘good luck charms’. This gruesome trade of human beings’ body parts sees heightened activity at certain times, such as elections, with those in potential power seeking ‘luck’ with the outcome [40].

Segregation of people with albinism begins early in their life [17]. In 2001, Lund [36] reported on a study conducted in Zimbabwe where children with albinism were found to be feared and ostracised. In our study, participants’ stories captured the impacts of albinism on childhood, with many children’s lives blighted as a result of such fear and rejection, often by their own families. Mothers of children with albinism are often accused of infidelity and blamed for their child’s condition. Previous studies have highlighted the significant problem of paternal abandonment whereby many fathers–and sometimes mothers—reject children with albinism [13, 16]. We heard some examples of couples loving and staying with their child with albinism. These narratives run contrary to those of rejection, but they are the minority. When understanding the lives of children with albinism, abandonment remains a problem. The consequences are that many children with albinism are raised in families where one or sometimes both parents have abandoned them, creating financial difficulties that impact negatively on education and health [14].

Lynch et al. [41] investigated strategies to improve the educational inclusion of visually impaired children with albinism in Malawi. Findings showed that children had heard about the myths surrounding albinism and this created anxiety. Like our study, previous studies have identified how teachers are afraid of children with albinism [17, 42]. However, we have been able to highlight that teachers can be crucial in challenging stereotypes and stigma and advocating for children with albinism. This is reliant however, on them first overcoming their own judgements, so that they can act as powerful role models. Their role in this respect has been associated with increased self-esteem and sense of belonging among children [15].

Our study findings highlight the significant disadvantage that many people with albinism experience as they progress into adolescence and adulthood, where discrimination and prejudice bar their access to higher education and employment. Again, sadly, this concurs with evidence spanning the past 20 years [11, 15, 16, 17, 43]. However, it is important to bring to the fore a counter-deterministic narrative of resilience and empowerment. In the context of Ubuntu, Metz and Gaie [44] assert that one’s ultimate goal is to become a ‘full person’ [44]. For people with albinism, this means building resilience to the extremely powerful discourse of them being ghosts, rather than ‘real’ people. In the context of our study, some people with albinism talked of overcoming struggles at school and work, achieving success and acting as powerful role models and advocates. Such success was considered an act of deity, with God giving them personal strength. It is important to emphasise however, that every person with albinism who took part in the study talked of discrimination against them and frequently cited incidents of abuse and violation.

As regards points for action, advocacy groups such as SNUPA are known to have a role in influencing community and government action [15] and education of people with albinism, their families, communities and traditional healers is important [36]. These mechanisms can be used as conduits for information about albinism that respects African knowledge systems and Ubuntu philosophies, while dispelling harmful myths.

### Limitations

The principal methodological limitation ironically arises from the success of the study in terms of how many people wanted to take part. Sample size in qualitative research is contentious, with much debate about the point at which data saturation is likely to occur [45, 46]. Using data from a study involving sixty in-depth interviews with women in two West African countries, Guest et al. [47] systematically documented the degree of data saturation and variability over the course of their thematic analysis. They analysed the 60 interviews in batches of six. Eighty codes were identified in the first six transcripts, with 20 more added in the second batch. Thereafter, new codes were almost non-existent leading the research team to conclude that data saturation occurred by the time of the twelfth interview.

Our sample size of 73 reflects a highly successful recruitment strategy and is indicative of how keen people affected by albinism (both directly and indirectly) are to talk about the issue in Uganda, where this is the first study of its kind. Yet this exceptionally large qualitative sample size has been a double-edged sword. On one hand it has allowed us to generate a dataset that contains rich information about the lives of people with albinism and the beliefs and myths surrounding their condition. This adds significantly to the extant body of knowledge in this hitherto largely neglected area of empirical investigation. On the other hand, it has been difficult to manage such an expansive set of data.

We have debated often how to do justice to the many accounts of those who took part, which quotes to include, whose voices to privilege, etcetera. Ethically we feel a responsibility to ensure that the findings are reported sufficiently. We have reconciled this through the secure archiving of data that can lend itself to future analysis as appropriate. Our focus in this article has been to report on the beliefs and myths about albinism that coalesce around the domains of CSM as the theoretical framework. Accepting the inherent incompleteness of this, does not undermine the value of the article. Instead it invites further interrogation of the data to unearth other important aspects of this important topic. Moreover, given that the study was limited only to people in families and communities close to people with albinism, new studies may be useful in understanding wider societal beliefs about the condition.

### Conclusions

Existing literature on the experiences of people with albinism is sparse, particularly that arising from empirical study, but overall our findings align with available evidence. This is not the first article about albinism and beliefs, but it is the first to organise such beliefs around a framework (CSM), so that they can be articulated clearly. It is also original in situating that framework within the context of Ubuntu. This is important because the beliefs are part of an African knowledge system that may be difficult for many people to understand.

Baker and colleagues [17] argued that widely held myths are one of the greatest hindrances to people with albinism taking full part in society, achieving personal growth and being accepted socially. While our study has some messages of hope and personal strength among some people with albinism, it is important to view this in a broader context. Most people with albinism endure life-long, systemic discrimination and violence. Even those who manage to overcome social and economic barriers, still encounter actual or potential risk on a day-to-day basis. Thus, there is still a great deal to be done. Our study contributes to this by providing a framework through which beliefs and misbeliefs can be understood so that in turn, the latter can be challenged and dispelled.
